# L-DIG: A GAN-Based Method for LiDAR Point Cloud Processing under Snow Driving Conditions

**DOI:** 10.3390/s23218660

**Published:** 2023-10-24

**Authors:** Yuxiao Zhang, Ming Ding, Hanting Yang, Yingjie Niu, Yan Feng, Kento Ohtani, Kazuya Takeda

**Affiliations:** 1 Graduate School of Informatics, Nagoya University, Furo-cho, Chikusa-Ward, Nagoya 464-8601, Japan; 2 Institutes of Innovation for Future Society, Nagoya University, Furo-cho, Chikusa-Ward, Nagoya 464-8601, Japan; 3 Tier IV Inc., Nagoya University Open Innovation Center, 1-3, Mei-eki 1-chome, Nakamura-Ward, Nagoya 450-6610, Japan

**Keywords:** LiDAR point cloud processing, snow noise removal, snow effect generation, CycleGAN

## Abstract

LiDAR point clouds are significantly impacted by snow in driving scenarios, introducing scattered noise points and phantom objects, thereby compromising the perception capabilities of autonomous driving systems. Current effective methods for removing snow from point clouds largely rely on outlier filters, which mechanically eliminate isolated points. This research proposes a novel translation model for LiDAR point clouds, the ‘L-DIG’ (LiDAR depth images GAN), built upon refined generative adversarial networks (GANs). This model not only has the capacity to reduce snow noise from point clouds, but it also can artificially synthesize snow points onto clear data. The model is trained using depth image representations of point clouds derived from unpaired datasets, complemented by customized loss functions for depth images to ensure scale and structure consistencies. To amplify the efficacy of snow capture, particularly in the region surrounding the ego vehicle, we have developed a pixel-attention discriminator that operates without downsampling convolutional layers. Concurrently, the other discriminator equipped with two-step downsampling convolutional layers has been engineered to effectively handle snow clusters. This dual-discriminator approach ensures robust and comprehensive performance in tackling diverse snow conditions. The proposed model displays a superior ability to capture snow and object features within LiDAR point clouds. A 3D clustering algorithm is employed to adaptively evaluate different levels of snow conditions, including scattered snowfall and snow swirls. Experimental findings demonstrate an evident de-snowing effect, and the ability to synthesize snow effects.

## 1. Introduction

**Figure 1:**
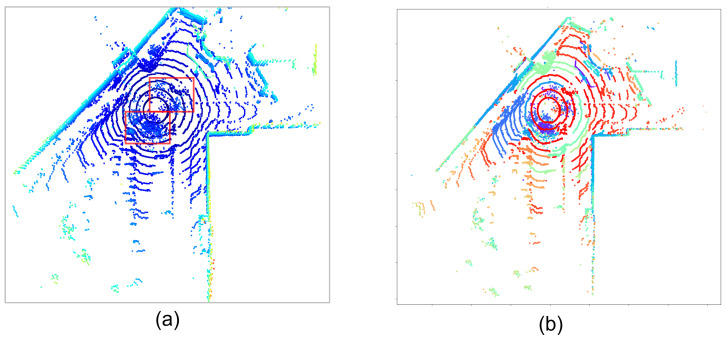
A frame of the point cloud featuring both dispersed noise points and snow clusters. (**a**) The original point cloud from the Canadian Adverse Driving Conditions (CADC) dataset [2], with colors representing height—the lighter the color, the greater the distance from the ground. Red boxes annotate scattered snow and snow swirl points. (**b**) The clustering result of the same point cloud based on the OPTICS (Ordering points to identify the clustering structure) algorithm, where varying colors signify different cluster groups. Objects and structures with regularized forms are aptly segmented into large-scale clusters, while scattered snow noise points constitute smaller clusters with minimal points. The prominent purple cluster at the center represents the snow swirl.

The presence of weather conditions affects and corrupts the signal quality of autonomous driving system (ADS) sensors and causes perception failures [1]. During the past decade, with the expansion of weather-related datasets [2,3] and the development of sophisticated machine learning techniques, models designed to address adverse weather problems, such as precipitation, in autonomous driving have been widely studied [4]. Progress has been made in the weather models of both LiDAR and images with weather conditions commonly treated as uniformly or normally distributed noise that can be represented by linear or monotone functions [5,6]. Snow is among the conditions with tangible threats to the sensors but few profundities in research. What makes it even more special is the unexpected irregular swirls phenomena brought by wind or the motions of the ego vehicle itself or passing-by vehicles [7], causing not only randomly distributed scattered noise (salt-and-pepper) but also swirl clusters in LiDAR point clouds, as shown in the disordered clusters near the center in the red boxes of Figure 1a. The study on the snow problem in the point cloud has been focusing on k-d-tree-based neighbor searching outlier filters [8] in recent years and the de-noising performance has almost reached saturation [9]. Nonetheless, few attempts at the implementation of deep-learning-based models have been made in snow conditions. Unlike filters with limited explainability, learning-based models have the potential to grasp both the surface and hidden features of snow clusters in a certain driving scene and perform snow synthesizing on top of snow de-noising.

The development of robust weather models benefits from training on paired data, i.e., a pair of weather-corrupted data and clear data with the rest of the elements identical, which are commonly obtained via artificially synthesizing realistic weather effects in previously clear driving scene images [10,11,12]. Such an approach has been proven highly effective in rain [13,14], fog [15,16], and snow [17] weather conditions in camera images, plus contaminations on the camera lens [18]. However, due to the relatively low data density, the realization of the weather effects in point clouds still largely depends on the collections in weather chambers [19,20] before the mature realization of weather data augmentation in point clouds. Although models have been successfully built for point clouds under rain and fog with additional road data [21], the low domain similarities between chambers and real roads still largely limit the generality. What is more, common experimental facilities with controllable precipitation rates across the world can hardly simulate complicated weather conditions such as dynamic snowfall [4]. Therefore, it is necessary to develop a way to work with few paired data or unpaired data.

In terms of disentangled data processing, CycleGAN [22] demonstrates a high ability in style conversion and object generation [23] based on datasets with different backgrounds and from different domains, and its implementation in weather models has been proven feasible [17,24]. In this research, we propose the ‘L-DIG’ (LiDAR depth images GAN), a GAN-based method using depth image priors for LiDAR point cloud processing under various snow conditions. The original data format of the LiDAR point cloud in the spatial dimension does not align with the plane dimension as camera images, while a depth image is able to store the third dimension information of a point’s spatial coordinates into its pixel value, hence serving as the 2D representation of the LiDAR point cloud, providing the opportunity of employing GAN-based methods [25]. Our unpaired training datasets are derived from the Canadian Adverse Driving Conditions (CADC) dataset [2], which is known for its authentic winter driving scenarios and snow diversity. The proposed model aims to perform snow de-noising with a deep-level understanding of the snow features related to the driving scene, and inversely generate fake snow conditions in clear point clouds, exploring the possibility of creating paired synthetic datasets for point clouds with snow conditions.

Furthermore, the quantitative evaluation of LiDAR point cloud processing has always been a tricky task. Researchers used to select a certain amount of samples, 100 frames for example, and manually determine if a point is a snow point, in order to calculate the precision and recall of the removal of snow noise points [8,26]. Even though straightforward, it has two downsides: For one, it consumes an unimaginable amount of time and manpower to manually annotate a whole dataset while a small portion of samples suffers the risk of bias. Secondly, the accuracy of human annotation on point clouds with over 20,000 points in each frame can be as low as 85% and not satisfied enough to support the subsequent calculations on precision and recall [27]. Given that a LiDAR point cloud fundamentally represents a distribution of points within a three-dimensional space, 3D point clustering algorithms can be effectively used to reflect the physical properties and assess the performance of point cloud processing specifically on snow points [28].

Among various algorithms, the *ordering points to identify the clustering structure* (OPTICS) algorithm [29] emerges as a preferred choice due to its exceptional capacity in handling clusters of varying densities [30]. Derived from the DBSCAN (density-based spatial clustering of applications with noise) methodology, OPTICS identifies distinct cluster groups by densely associating data points within a predetermined radius [31]. This principle highly aligns with the adaptive filters, which are predicated on neighbor searching and have been widely employed in the LiDAR point cloud de-noising over recent years.

An example of the OPTICS clustering result is shown in Figure 1b. It can be seen that despite the density variation, everything has been classified into separated groups of clusters, each distinguished by unique colors, while the environmental structures are also well segmented. The conglomeration of snow swirl points, positioned at the lower left of the center, are collectively assigned to large blue and purple clusters. Minor snow clusters, such as those in the immediate right vicinity of the center, along with individual scattered snow points spread across the scene, are categorized into smaller, uniquely colored clusters.

The main contributions of this research are described as follows:

1. We build a deep-learning-based LiDAR point cloud translation model with unpaired data and depth image priors. A new discriminator structure to better remove snow noise and new loss functions, including depth and SSIM (structural similarity index measure) losses to maintain the driving scene integrity have been designed in the proposed model.
2. The proposed model is able to perform LiDAR point cloud translations between snow conditions and clear conditions in driving scenes. The model demonstrates a certain level of understanding of the snow features and performs both effective snow de-noising and artificial snow point generation, which could help create paired datasets for training or simulation in autonomous driving applications.
3. We employ the OPTICS clustering algorithm as the quantitative evaluation metrics of the snow conditions in LiDAR point clouds. We set adaptive parameters to cluster different forms and levels of snow masses, calculate multiple indexes, and present distribution changes in violin plots to reflect the conditions of snow in the whole dataset in a comprehensive way.

## 2. Related Works

Our proposed model is a GAN-based deep-learning model for LiDAR point cloud processing including de-noising. As a result, we are going to introduce some related works regarding adaptive noise removal filters, deep-learning-based LiDAR point cloud processing methods, and GAN-based LiDAR translation methods in the following sections.

### 2.1. Adaptive Noise Removal Filters in LiDAR Point Cloud Processing

Noise point removal, or to say de-noising, is one of the most fundamental preliminary tasks of LiDAR point cloud processing to enhance the data quality and is commonly realized by radius outlier removal (ROR) filters. In the 3D space, ROR filters methodically analyze each individual point, assessing neighboring points located within a predefined radius. If the count of these neighboring points is below a certain threshold ($k_{min}$), the filter deems the point as noise and eliminates it. This approach is notably effective in removing noise generated by small, separate solid entities such as snowflakes. However, it carries a potential drawback as it might also eliminate distant points in the environment, thus compromising the data integrity of the original point clouds. One of the main reasons for this limitation lies in the mismatch between the fixed predefined search radius and the varying point density that tends to decrease towards the edges of the point clouds. As a result, there is a compelling demand for an adaptive search radius to rectify this discrepancy.

Charron et al. [8] proposed an advanced variant of the conventional filter, referred to as the dynamic radius outlier removal (DROR) filter. The distinctive characteristic of the DROR filter is that it dynamically adjusts the search radius ($SR_{p}$) of each point based on its intrinsic geometric attributes, as shown in (1), where $r_{p}$ is the range from the sensor to the point *p*, α is the horizontal angular resolution of the LiDAR, the product of $\left(r_{p}\alpha \right)$ represents point spacing, and $\beta_{d}$ is the multiplication factor. This approach meticulously eliminates central noise points, while facilitating the retention of pivotal points situated at greater distances within the point clouds, successfully preserving the pertinent structures in distance.

(1) $$SR_{p}=\beta_{d}\left(r_{p}\alpha \right)$$

Another noteworthy innovation is the dynamic statistical outlier removal (DSOR) filter introduced by Kurup and Bos [26]. In this approach, point removal involves a thorough examination of each point to ascertain if the mean distance to its k nearest neighbors exceeds a dynamic threshold. The calculation of this dynamic threshold is defined as follows:

(2) $$T=\mu +\sigma \beta_{s}$$

(3) $$T_{d}=r\left(Tr_{p}\right)$$

where the variables are designated as follows: μ symbolizes the overall mean distance between every point and their corresponding *k* nearest neighbors. The global standard deviation of these distances is represented by σ. The term $\beta_{s}$ stands for a predetermined multiplier parameter. The distance from the sensor to a given point *p* is indicated by $r_{p}$, whereas *r* signifies a scaling factor for the spacing between points. In terms of performance, DSOR proves to be either on par with or superior to DROR in both noise reduction and environmental feature preservation. Remarkably, DSOR also exhibits a significantly higher computational speed compared to DROR, further underlining its advantages in data preparation tasks.

Subsequently, a variety of optimized filters based on adaptive parameters such as intensity-based [32], and density-based [33] were put forward. While adaptive filters have made strides in de-noising, they are inherently constrained by predefined removal rules. This limitation becomes particularly evident when considering the diverse and infinite ways that snow can form clusters, a complexity that cannot be fully captured by such rigid algorithms.

### 2.2. Deep-Learning-Based LiDAR Point Cloud Processing

The initial implementation of point cloud de-noising using deep learning was primarily focused on mitigating the effects of rain and fog, which introduce obscurity or diffusion of LiDAR signals due to the presence of small water droplets. Additionally, external disturbances such as wind or spray can lead to the formation of clustered fog and water mist, thereby causing false obstacles for LiDAR [34]. Shamsudin et al. [35] developed algorithms for removing fog from 3D point clouds after detection using intensity and geometrical distribution to separate and target clusters of points, which were then removed from the point cloud. The problem is such algorithms are designed for indoor environments where fog behaviors are significantly different than outdoor driving scenarios.

To address the weather problems under driving conditions, Heinzler et al. [21] proposed a CNN-based approach capable of weather segmentation and de-noising. The authors collected both clear and foggy datasets within the CEREMA’s climatic chamber [20], enabling controlled precipitation rates and visibility conditions. By utilizing the paired datasets, they facilitated an automated labeling procedure that annotated weather data, enabling the model to acquire knowledge regarding the distinctive attributes of rainfall and fog reflections in point clouds. Subsequently, the trained model demonstrated the ability to discriminate fog/rain-induced point clusters and eliminate noise while preserving the original objects, such as pedestrians or cyclists. To enhance the model’s capability of comprehending real-world elements that are challenging to simulate in fog chambers, the authors also incorporated fog and rain data augmentation upon road data in the CNN model.

Besides de-noising techniques, augmentation is another valuable LiDAR point cloud processing method that can offer comparable assistance in managing adverse weather conditions. Hahner et al. [36] developed a method that simulates snow particles in a 2D space corresponding to each LiDAR line and adjusts each LiDAR beam’s measurements based on the resulting geometry. In addition, they factored in ground wetness, a common occurrence during snowfall, in their LiDAR point clouds as a supplement of the augmentation. The notable enhancement observed in the performance of 3D object detection subsequent to training on semi-synthetic snowy training data substantiates the successful simulation of snowfall. It is of particular importance to acknowledge that their snow augmentation approach predominantly focuses on light snowfall conditions under the rate of 2.5 mm/h, wherein the prevalent snow effects in LiDAR point clouds manifest as dispersed noise points rather than snow clusters. Snow clusters pose a greater challenge to LiDAR perception in actual driving conditions and our primary focus revolves around the study of snow clusters.

### 2.3. GAN-Based LiDAR Translation among Various Adverse Conditions

The utilization of GANs is particularly suitable for weather de-noising tasks due to the inherent challenge of obtaining diverse weather conditions while maintaining a consistent background at the pixel level, given the ever-changing atmospheric conditions. Notable methods in this area include CycleGAN [22], DiscoGAN [37], and DualGAN [38], which introduce a cycle-consistency constraint to establish connections between the images. Leveraging the capabilities of GANs to generate visually realistic images without requiring paired training data, translations between weather images and clear ones have been widely studied. De-weather frameworks based on GAN structure have proven effective in removing multiple weather conditions [24,39] in a single image.

Consequently, the adaptation of GAN models for the translation of LiDAR point clouds has emerged as a natural progression. Sallab et al. [40,41] were the first to explore the translation from simulated CARLA driving scenes to synthetic KITTI [42] point clouds, employing the CycleGAN framework. Similarly, Lee et al. [43] turned to point cloud translations between sunny, rainy, and foggy weather conditions. They employed the depth and intensity channels of the 2D Polar Grid Map (PGM) for CycleGAN processing. It is worth noting that inter-domain translations between fog chamber scenes and real-world roads present a challenge wherein artificial precipitation from sprinklers can be detected as vertical cylinders rather than genuine rainfall by LiDARs [1]. This discrepancy may impact the interpretability of weather reflection features in point clouds, thereby potentially diminishing the overall translation performance. The inability of chambers to simulate snowy conditions has also resulted in a lack of advancements in the processing of LiDAR point cloud data in snow environments.

## 3. Methods

### 3.1. LiDAR 2D Representation

To adapt LiDAR point cloud data to fit within the structure of the GAN-based model, we initially applied a dimensionality reduction to the point clouds. This yields a 2D visualization of the point clouds, namely, depth images, which signifies the orthographic projection of the point clouds. By unrolling the exterior surface of the LiDAR’s horizontal field of view (FOV) cylinder and mapping each point from the point cloud onto this frontal-view plane, a rectangular image encompassing all points within the LiDAR’s FOV is obtained. We partition the horizontal field evenly into *w* columns and distribute the vertical field uniformly into *h* rows. Consequently, post-projection, a depth image bearing a resolution of *w* × *h* can be secured, where the horizontal resolution is proportional to the sensor’s rotation rate, and the vertical resolution is proportional to the number of physical layers [44].

**Figure 2:**
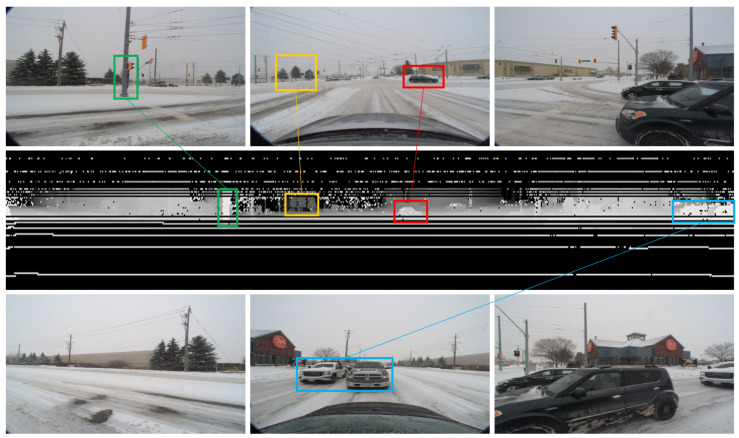
An illustration of a specific frame of the depth image under heavy snow conditions. The middle row displays the depth image, while the top and bottom rows depict corresponding RGB images derived from the CADC(Canadian Adverse Driving Conditions) dataset [2]. These images are captured from multiple cameras targeting different directions around the ego vehicle. Color-coded boxes indicate objects that are present in both the RGB and depth images. Green—A pole with a road sign and traffic lights. Yellow—Three trees. Red—A vehicle at the intersection. Blue—Two pick-up trucks waiting in line. Please note that the images provided do not represent the original resolutions; they have been adjusted to enhance their illustrative capacity.

An illustration of a specific frame of the depth images under noticeably snowy conditions, along with the corresponding RGB images featuring identical objects, can be found in Figure 2. A close observation reveals that the relevant objects are well reflected and the snow noises, bearing resemblance to ‘salt and pepper’ speckles, are prominently displayed in the depth image. The position of each pixel reflects the frontal-view projection of the points within the point clouds, while the grayscale pixel value signifies the distance between the points and the observing vehicle. Smaller pixel values (darker) imply greater distances, whereas higher pixel values (brighter) suggest closer distances. A discrepancy is worth mentioning that due to the limited vertical FOV and its relatively high mounting position on the car’s roof of the LiDAR used in the sample dataset, the black car in the immediate right vicinity of the ego vehicle partly falls within the “blind spot” and is not clearly delineated in the depth image. Instead, only a few high-intensity signal points from the window glass are visible.

To obtain depth images under clear conditions, we apply the DSOR filter [26] onto snow datasets. In this context, we invert the typical approach of creating synthetic weather conditions in datasets for training, instead of generating an artificial clear dataset. Given that filters cannot guarantee absolute precision and recall in the de-snowing process, the filtered result cannot be considered equivalent to the ground truth. However, the stringent filter still provides a valuable sample for unpaired training. Among the reasons we choose DSOR over alternative filters are its rapid processing speed and its excellent capacity to retain as many environmental elements as possible when the filter parameters are set to an uncompromising level.

### 3.2. LiDAR Depth Images GAN

**Figure 3:**
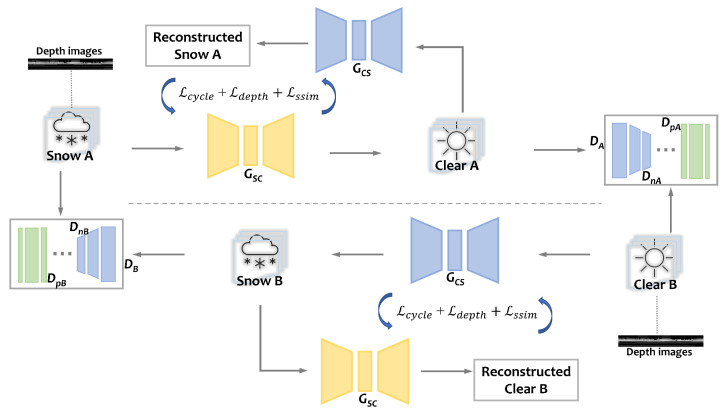
Proposed LiDAR translation model architecture. Datasets A and B are in the form of depth images. $G_{SC}$ and $G_{CS}$ are the generators. $D_{nA}$ and $D_{nB}$ are the N-layer discriminators, and $D_{p_{A}}$ and $D_{p_{B}}$ are the pixel-attention discriminators.

In this research, an enhanced iteration of CycleGAN [22] serves as the foundational structure for our proposed model, LiDAR depth images GAN (L-DIG). As illustrated in Figure 3, the comprehensive blueprint of our model is depicted. Due to the features of CycleGAN, both the de-snowing (snow→clear) and the fake snow generation (clear→snow) are completed at the same time for one set of data. Table 1 provides the annotations of all the symbols and alphabet designations used in the model architecture. In our model, A and B symbolize two sets of data flow in the forward and backward cycle, respectively, and Snow A and Clear B are the inputs that we provide in the form of depth images. *C* subscripts correspond to clear weather conditions, whereas *S* subscripts are used to indicate snowy conditions. The generators responsible for transitioning between snowy to clear and clear to snowy states are represented by $G_{SC}$ and $G_{CS}$, respectively. Meanwhile, the discriminators are expressed as $D_{A}$ and $D_{B}$. The two reconstructions are GAN features to keep the translation from overfitting. We provide the pseudo-code of L-DIG in Algorithm 1.

### 3.3. Pixel-Attention Discriminators

We designed a new discriminator structure to enable the model to recognize the snow noise points more accurately. The discriminators $D_{A}$ and $D_{B}$ are each composed of two parts: N-layer Discriminators $D_{nA}$ and $D_{nB}$ with 3 convolutional layers, and pixel discriminators $D_{pA}$ and $D_{pB}$. The N-layer discriminators concentrate on relevant objects within the scene while the pixel discriminators scrutinize each pixel individually to ascertain its authenticity through a 1 × 1 patch [22]. This approach contributes to a minor disturbance to the binary discriminator’s threshold, elevating the criteria to achieve a 1 (approved) rather than a 0 (rejected) to a more stringent level for isolated noise points. This strategy markedly enhances the de-snowing effect, particularly in areas surrounding the ego vehicle, where dispersed snow points are densely packed. The pixel-attention discriminators undergo training for several epochs subsequent to the stabilization of the model training with N-layer discriminators, as shown in Figure 3 and Algorithm 1.
**Algorithm 1** LiDAR Depth Images GAN (L-DIG)**Require:** Training data pairs (A,B)    ⊲ Snow A and Clear B**Ensure:** Generator networks $G_{SC}$ and $G_{CS}$, N-Layer Discriminators $D_{n_{A}}$, $D_{n_{B}}$, and Pixel Discriminators $D_{p_{A}}$, $D_{p_{B}}$ 1:Initialize generators $G_{SC}$, $G_{CS}$ and N-Layer Discriminators $D_{n_{A}}$, $D_{n_{B}}$ 2:Define loss functions including GAN, cycle, depth, and SSIM loss 3:Define optimizers for generators and discriminators 4:**while** epoch ≤ (total_epochs - continued_epochs) **do** 5:   **for** each data pair (A,B) in data_loader **do** 6:     Generate fake images: $F_{A}=G_{CS}\left(B\right)$, $F_{B}=G_{SC}\left(A\right)$ 7:     Generate reconstructed images: $Rec_{A}=G_{CS}\left(F_{B}\right)$, $Rec_{B}=G_{SC}\left(F_{A}\right)$ 8:     Compute GAN, cycle, depth, and SSIM loss 9:     Update discriminators $D_{n_{A}}$, $D_{n_{B}}$ and generators $G_{SC}$, $G_{CS}$10:   **end for**11:**end while**12:Initialize Pixel Discriminators $D_{p_{A}}$, $D_{p_{B}}$13:**while** (total_epochs - continued_epochs) < epoch ≤ total_epochs **do**14:   **for** each data pair (A,B) in data_loader **do**15:     Use the same generators to produce fake images as in previous training16:     Compute GAN, cycle, depth, and SSIM loss17:     Update discriminators $D_{p_{A}}$, $D_{p_{B}}$, $D_{n_{A}}$, $D_{n_{B}}$ and generators $G_{SC}$, $G_{CS}$18:   **end for**19:**end while**

### 3.4. Loss Function

#### 3.4.1. Depth Loss

Scale ambiguity poses a challenge for depth images, necessitating the use of loss functions resilient to rough estimations [45]. Taking a cue from the *Fine* network [46], we crafted a depth loss, $L_{depth}$, that is integrated into the training cycles to uphold consistency in the scale of depth images, as represented in (4).

(4) $$L_{depth}=\frac{1}{n}\sum_{i}{\left(\hat{d_{i}}-d_{i}\right)}^{2}-\frac{\lambda_{depth}}{n^{2}}{\left(\sum_{i}\left({\hat{d}}_{i}-d_{i}\right)\right)}^{2}$$

In the given formula, $\hat{d_{i}}$ and $d_{i}$ symbolize the reconstructed and initial depth, respectively, while the hyperparameter $\lambda_{depth}$ governs the scale invariance. We assign $\lambda_{depth}=1$ to achieve complete scale invariance. This is due to our objective of preserving the translated point cloud as similar to the original as possible, exclusive of the snow, and safeguarding the relevant objects and environmental elements from distortions in shape and size.

#### 3.4.2. SSIM Loss

As the point cloud translation occurs on the scale of the entire scene, it sometimes involves objects and structures that are partially obscured or incomplete, leading to the model’s suboptimal comprehension of these elements. This can cause distortions or alterations in the original forms, particularly in environmental features. To mitigate this, an SSIM (structural similarity index measure) loss, depicted in (5) and (6), has been incorporated into the training cycle to aid in preserving structural consistency.

(5) $$SSIM\left(N,\hat{N}\right)=\frac{\left(2\mu_{N}\mu_{\hat{N}}+c_{1}\right)\left(2\sigma_{N\hat{N}}+c_{2}\right)}{\left(\mu_{N}^{2}+\mu_{\hat{N}}^{2}+c_{1}\right)\left(\sigma_{N}^{2}+\sigma_{\hat{N}}^{2}+c_{2}\right)}$$

(6) $$L_{ssim}=1-SSIM\left(\hat{N},N\right)$$

where N is the normalized image tensor of the original real image, $\hat{N}$ is the normalized image tensor of the reconstructed image, $\mu_{\hat{N}}$ is the average of $\hat{N}$, $\mu_{N}$ is the average of *N*, $\sigma_{\hat{N}}^{2}$ is the variance of $\hat{N}$, $\sigma_{N}^{2}$ is the variance of *N*, $\sigma_{\hat{N}N}$ is the covariance of $\hat{N}$ and *N*, c1 and c22 are two variables to stabilize the division with a weak denominator.

The SSIM loss computation is performed post a subtraction by 1, due to the fact that SSIM loss gauges similarity while the training mechanism is geared towards attaining minimum values. Consequently, we aim to lower the difference by training 1 minus the SSIM function. This also elucidates the necessity for prior normalization on the image tensor between [0, 1]. Meanwhile, it is essential to maintain a relatively low $\lambda_{s}$ weight setting on the SSIM loss to prevent the model from becoming overly rigid, thereby obstructing any desired translation.

#### 3.4.3. Cycle Consistency Loss

We employ the cycle consistency loss (7) derived from CycleGAN, aimed at maintaining the created depth images closely aligned with the original domain. Provided a minimal variation in the background during the translation, the weight $\lambda_{c}$ of cycle consistency loss can be set as equal to the customized depth loss.

(7) $$\begin{array}{cc} L_{cyc} & =\|G_{SC}\left(G_{CS}\left(S_{B}\right)\right)-S_{B}\left\|+\right\|G_{CS}\left(G_{SC}\left(C_{A}\right)\right)-C_{A}\| \end{array}$$

#### 3.4.4. Overall Loss Function

Upon integrating the conventional GAN adversarial losses between the clear and snowy data, denoted by $L_{GAN}\left(G_{SC},D_{A},S,C\right)$ and $L_{GAN}\left(G_{CS},D_{B},C,S\right)$, we arrive at the comprehensive objective loss:

(8) $$\begin{array}{cc} L(G_{SC},G_{CS},D_{A},D_{B}) & =L_{GAN}\left(G_{SC},D_{A},S,C\right)+L_{GAN}\left(G_{CS},D_{B},C,S\right)\\ & +\lambda_{c}L_{cyc}+\lambda_{d}L_{depth}+\lambda_{s}L_{ssim} \end{array}$$

where $\lambda_{c}$, $\lambda_{d}$, and $\lambda_{s}$ denote the weight coefficients of cycle consistency loss, depth loss, and SSIM loss, respectively. The higher the weight, the larger the influence the corresponding loss function has on the model.

### 3.5. OPTICS 3D Clustering Algorithm

OPTICS exhibits great adaptability to variable densities, presents lower parameter sensitivity, exhibits hierarchical relationships among clusters, and possesses improved outlier detection capabilities. These features of OPTICS largely match with snow point behaviors in point clouds and make it a nice evaluation method on snow datasets. By applying an adaptive clustering principle, we significantly simplify the interpretation and extraction of clusters without calling excessive manual parameter tuning.

We produce the following seven metrics based on the OPTICS algorithm:

- Noise Number: Points without any neighbor points within a designated range (solitary points) are considered noise points, mostly snowflakes. A decrease in noise number is one of the most direct indicators of an effective de-snowing performance.
- Cluster Number: A main output of the algorithm, representing groups of data points that are closely related based on their reachability.
- Reachability Distance: The smallest distance required to connect point A to point B via a path of points that satisfy the density criteria. Normally, the average reachability distance would rise along with larger cluster numbers.
- Inter-Cluster Distances: The concept here involves identifying the centroid, or the average point, of each cluster, and subsequently computing the distance between every possible pair of centroids. Should there be an increase in the average of these distances, it would suggest a reduction in the number of clusters and a more dispersed cluster distribution. In the context of our study, such a pattern could be interpreted as an effect of de-snowing.
- Size of Clusters: This is essentially determined by the average number of points each cluster holds. Under conditions dominated by scattered snow, the snow noise points tend to form numerous small-scale clusters. Their elimination, consequently, leads to an increase in the average size of the clusters.
- Silhouette Score: Measures the cohesion within clusters and the separation between clusters. A silhouette score close to 1 indicates a good clustering quality, while a score close to −1 indicates poor clustering. A lower silhouette score is commonly observed in snowy conditions due to the more overlap between clusters.
- Davies-Bouldin Index (DBI): Measures the ratio of within-cluster scatter to between-cluster separation and assesses the quality of the overall cluster separation. A lower Davies-Bouldin index indicates better clustering, with zero being the ideal value. Snow conditions with many noise points or swirl clusters exhibit higher values of DBI.

**Figure 4:**
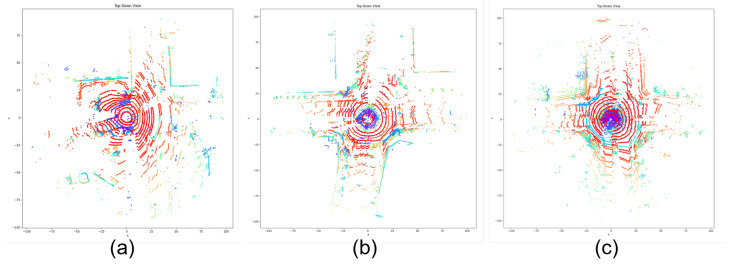
The clustering results of three example scenes from the OPTICS algorithm, where varying colors signify different cluster groups. (**a**) Slight snowfall condition with bits and pieces of snow points. (**b**) Normal snowfall condition with both scattered snow points and snow clusters around the center. (**c**) Fierce snow swirl condition with huge snow swirl clusters surrounding the ego vehicle.

We present three example scenes in Figure 4, from a slight snowfall condition to a normal snowfall condition, as well as a fierce snow swirl condition to illustrate how the OPTICS algorithm evaluates snow conditions, with their metrics summarized in Table 2. From the statistics, we can tell that with the increase in snow level, the noise number and cluster number of the driving scene are gradually rising, along with the average reachability distances as expected. With fewer noise points and fewer snow clusters, the average inter-cluster distances and the average sizes of clusters decrease correspondingly. The tendencies toward deteriorated clustering and increased overlaps, as indicated by the DBI and silhouette score, are also consistent with conditions of heavier snow conditions. This validation proves the OPTICS algorithm’s capability of evaluating the change of snow conditions in a LiDAR point cloud.

**Figure 5:**
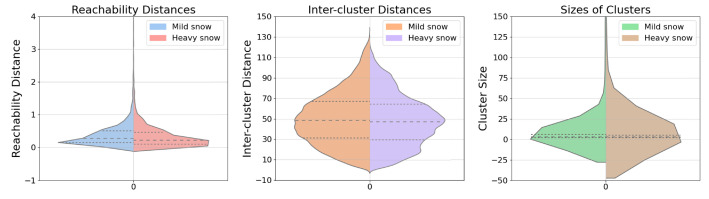
Violin plots for the comparison between mild snow and heavy snow. Limits on the y-axes are set for a better illustration of the distributions.

In order to more effectively illustrate the differences between mild and heavy snow conditions in terms of reachability distances, inter-cluster distances, and cluster sizes, we provide violin plots for these three metrics in Figure 5, positioning mild snow on the left and heavy snow on the right in each set. These plots not only show their quartiles but more crucially, delineate the differences in distribution. It can be observed that as snow conditions get heavier, the distribution in all three metrics exhibits somewhat abrupt curves with sharper edges and sudden shifts, indicating the inherent disarray characteristic of heavy snow. Generally, scenes with less snow presence display a relatively uniform and smooth distribution, as depicted in the violin plots, with a lower skewness value [47], offering another angle to assess the changes in snow conditions.

## 4. Experiments and Results

### 4.1. Experiments

We conducted experiments with the trained models on two different conditions: (1) Mild snow conditions: snowfalls only without snow swirls; (2) Fierce snow conditions: both snowfalls and snow swirls. We first conducted the experiment on snowfall-only conditions to examine the performance of scattered noise point capture. In the meantime, this less occlusion condition provides a better opportunity to check how well the original environmental structures have been maintained, so as to affirm the model’s ability for accurate snow capturing. Then, the same experiment was conducted on conditions with both snowfalls and snow swirls, to comprehensively present the model’s ability to handle highly adverse conditions.

To guarantee the realism of the snow effect, we utilized the well-known dataset specializing in snow conditions within an autonomous driving context—the CADC dataset [2]. This dataset encompasses over 7000 frames of LiDAR point cloud data gathered during the winter season in Waterloo, Ontario. The driving scenarios span both urban and suburban settings, incorporate high and low-speed conditions, and cover a range of snowfall intensities and heavy snow accumulation situations. The LiDAR system implemented in the CADC dataset has a vertical FOV of 40${}^{\circ }$, with a range from +10${}^{\circ }$ to −30${}^{\circ }$, and a horizontal FOV of 360${}^{\circ }$.

Training, testing, and data processing were conducted utilizing the Pytorch framework. We initially examined all possible combinations of ResNet residual blocks (ranging from 4 to 9) in $G_{SC}$ and $G_{CS}$, and convolutional layers (ranging from 1 to 4) in $D_{nA}$ and $D_{nB}$, and identified the most optimal combination for our model. When variables are kept constant, a combination of four ResNet residual blocks in $G_{CS},G_{SC}$ and two downsampling convolutional layers in $D_{nA}$ and $D_{nB}$ produce the most superior translation result.

Square-shaped samples randomly cropped from the depth images are input to two NVIDIA RTX 3090Ti graphics cards with a batch size of eight for training. In the second half of the N-Layer discriminator stage training, we adhere to a linearly declining learning rate schedule until the process converges.

The quantitative analysis is conducted based on 500 samples under mild snowfall conditions and the other 500 samples under fierce snow swirl conditions out of the testing dataset. The reported metrics in the following results all mean the average values.

### 4.2. Results

#### 4.2.1. Mild Snow Conditions

**Figure 6:**
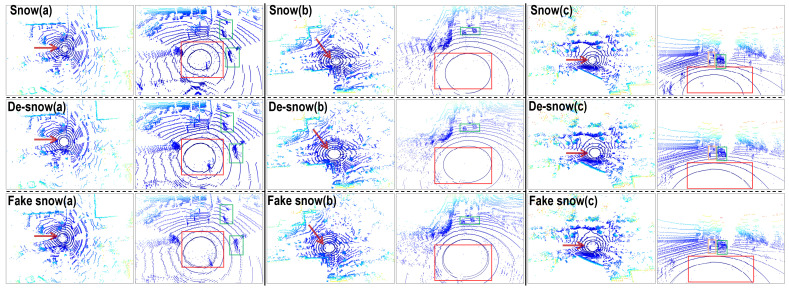
Qualitative results of point cloud translations in scattered snow conditions (without snow swirls). Colors are encoded by height. The top row is the snow conditions from the CADC dataset, the middle row is our de-snowed results, and the bottom row is the fake snow results obtained from our model. Three scenarios are presented, with the left sides of each set being the overall BEV scenes and the right sides of each set being the enlarged third-person view center part around the ego vehicle. Red boxes and arrows denote the locations where snow’s effects are alleviated. Green boxes annotate vehicles. Orange boxes annotate pedestrians.

Figure 6 shows the translation results of our model under mild snow conditions, which means the majority of the snow is scattered noise points without the snow swirl phenomenon. (a), (b), and (c) sets show three typical scenarios with the top row being the original snow scene from CADC, the middle row being the de-snowed results, and the bottom row being the fake snow results. Each of the three scenarios presented features an overall BEV on the left, while the right shows a magnified third-person view of the point cloud’s central region, where the ego vehicle is situated.

As indicated by the red arrows and encircled by red boxes, it is clear that the ‘salt-and-pepper’ noise points have been largely erased, with key environmental features left unaltered. Essential components, like vehicles (outlined in green) and pedestrians (highlighted in orange), are not only well preserved but also exhibit a level of point enhancement, as demonstrated in the de-snow (a) set. Moreover, the road sign enclosed in the red box of (a) which was partially obscured by snow points in the earlier image, seems to be better defined, a testament to the deep scene comprehension facilitated by our model.

The quantitative analyses are presented in Table 3. The noticeable reduction in the average noise number, cluster count, and overall reachability distances in the de-snowed results strongly suggests the effectiveness of the de-snowing process. As the majority of clusters now comprise object points and environmental features that are more densely and uniformly packed, the average inter-cluster distances, and average cluster sizes naturally increase. This shift in cluster characteristics is a byproduct of fewer, but more meaningful, clusters primarily representing substantive elements of the environment rather than scattered snow points. Similarly, the declines in the DBI and silhouette score are in line with our expectations for the de-snowing process.

**Figure 7:**
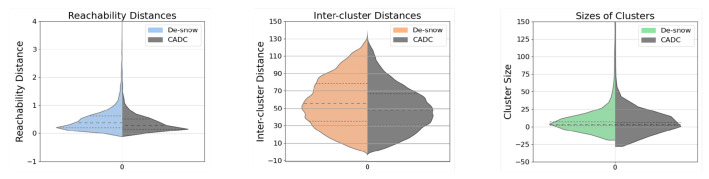
Violin plots for **de-snow** results under mild snow conditions. Limits on the y-axes are set for a better illustration of the distributions.

In the violin plots of Figure 7, the colored data on the left represents de-snowed data, while the gray data on the right serves as a comparison from the CADC dataset. This arrangement is consistent across all subsequent violin plots. A glance at the better evenness within the cluster distribution on the left half of each violin plot reveals the improvement of the de-snowing process compared to the slightly skewed distribution on the right. This observation is further substantiated by the lower skewness of the de-snowed distributions. Our calculations show that for the reachability distances, inter-cluster distances, and sizes of clusters, the skewness values for the de-snow data are 8.11, 0.23, and 16.10, respectively, while for the CADC data, these values are 9.64, 0.30, and 21.49. Note that the median reachability distance of the de-snow is a little bit higher than with snow. This small anomaly originates from a few detached clusters at a remote distance after de-snowing, which can be seen from very few sample points exceeding the upper limit of the y-axis.

**Figure 8:**
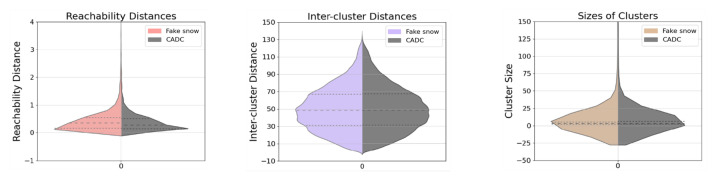
Violin plots for **fake snow** results under mild snow conditions. Limits on the y-axes are set for a better illustration of the distributions.

For fake snow generations, as seen in the bottom row of Figure 6, the scattered snow features are noticeably reproduced, and there is an apparent enhancement as the number of noise points is higher. This is in line with the noticeable increase in cluster number and DBI, as well as the reduction in cluster sizes, as presented in Table 3. The artificially generated snow demonstrates a remarkable replication capacity, as evidenced by the highly alike violin plots (left and right) in Figure 8, including the quartile lines. The degree of skewness (8.87, 0.33, and 22.43) is remarkably close to the previously mentioned CADC snow skewness (9.64, 0.30, and 21.49), further attesting to the model’s ability to accurately reproduce snow effects.

#### 4.2.2. Fierce Snow Conditions

**Figure 9:**
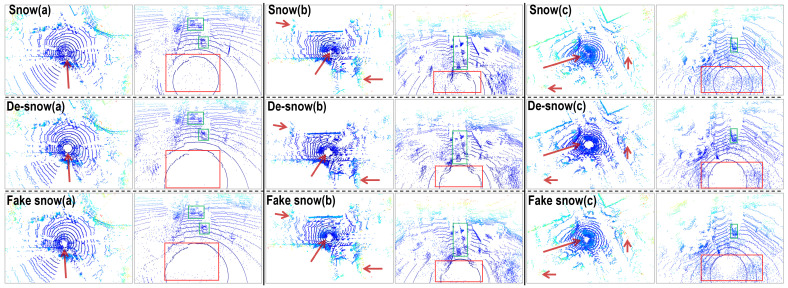
Qualitative results of point cloud translations in fierce snow conditions (with snow swirls). Colors are encoded by height. The top row is the snow conditions from the CADC dataset, the medium row is our de-snowed results, and the bottom row is the fake snow results obtained from our model. Three scenarios are presented, with the left sides of each set being the overall BEV scenes and the right sides of each set being the enlarged third-person view center part around the ego vehicle. Red boxes and arrows denote the locations where snow’s effects are alleviated. Green boxes annotate vehicles.

Figure 9 demonstrates the translation outcomes of our model under intense snow conditions, characterized by the presence of snow swirls around the ego vehicle. Three distinctive scenarios have been chosen for illustration, and are presented in the same format as in Figure 6. In these harsh snowy conditions where the snowfall has dramatically increased, it becomes easier to observe that the vibrantly colored airborne snowdrifts (highlighted in shades of red, green, yellow, and cyan) have been substantially mitigated, as indicated by the red arrows.

Under these severe snow circumstances featuring dense snow swirl clusters, our attention is more on the noise reduction near the ego vehicle, as indicated by the red boxes, instead of entirely eradicating the snow swirls, as this could lead to a loss of important environmental elements. We strive for a balance between significant snow removal and effective preservation of objects like the vehicles shown in the green boxes. Simultaneously, a certain degree of point cloud restoration can also be observed near the central ground rings. This can be credited to the profound comprehension of the scene by the translation model.

**Figure 10:**
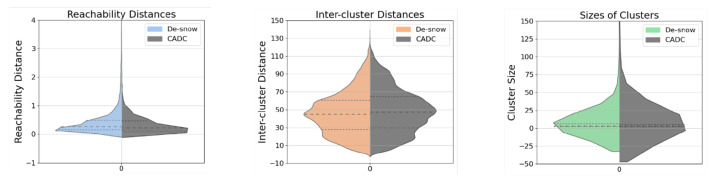
Violin plots for **de-snow** results under fierce snow conditions with swirls. Limits on the y-axes are set for a better illustration of the distributions.

Table 4 provides a quantitative representation of the translation model’s performance under extreme snow conditions. Given that the translation effects are applied to more clusters spanning the entire scene during heavy snowfall, all metrics largely veer towards less noise and tidier clustering results in the de-snowing task. From Figure 10, we can tell that the shifts in quartile lines are less prominent, which can be attributed to the fact that snow swirls typically have capacities similar to those of object clusters. Nevertheless, the efficacy of the de-snowing process is evidenced by the smoother and more consolidated distributions in the violin plots. This assertion is additionally validated by the slightly improved skewness of the de-snowed data which stand at 8.87, 0.38, and 28.04, respectively. Conversely, for the CADC data, these values are 10.45, 0.42, and 32.76.

**Figure 11:**
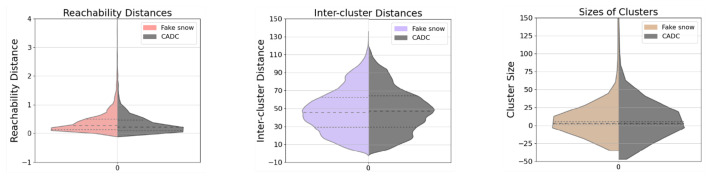
Violin plots for **fake snow** results under fierce snow conditions with swirls. Limits on the y-axes are set for a better illustration of the distributions.

As can be observed from the bottom row of Figure 9, our model effectively replicates airborne snowdrifts and the snow swirls surrounding the ego vehicle. However, the model exhibits a slight restraint at the point cloud’s central region. This outcome results from our strong focus on comprehending the driving scene, which is to avoid the broken integrity obstructed by the extremely dense snow swirl clusters. This situation leads to a somewhat compromised snow imitation, as corroborated by the close statistical outcomes in Table 4, which do not exhibit any significant jumps. Still, the near-symmetrical violin plots in Figure 11 further substantiate the successful emulation of snow effects. The smoother edges of reachability distances and the more concentrated distribution in cluster sizes of the imitation snow hint at a feature of reduced noise at the center. The skewness values associated with the artificially generated heavy snow are 9.95, 0.42, and 31.75, respectively. These values closely mirror those obtained from actual data, which are 10.45, 0.42, and 32.76, respectively. This statistical similarity provides additional evidence, affirming our model’s effectiveness in the task of synthetic snow generation.

### 4.3. Ablation Study

To affirm the significance of our model’s key components, we conduct an ablation study using the de-snow model under mild snow conditions. This study investigates the impact of the absence of the pixel-attention discriminator, SSIM loss, depth loss, and the basic CycleGAN. Additionally, we examine a training pair with a considerable domain gap. For this purpose, we select 6000 frames from the LIBRE dataset [1], which was collected under clear conditions in Nagoya, Japan’s urban area. This choice serves as a representative due to the substantial domain disparity between Canada and Japan in terms of scenario layouts and traffic patterns. The CADC dataset contains a large portion of suburban scenarios with fewer buildings and more vegetation, which hardly appears in the LIBRE dataset. Table 5 presents the results, using our proposed model as a reference.

The absence of the pixel-attention discriminator results in an immediate degradation in the performance, as evidenced by the increased noise number and reachability distance. Failing to remove a certain amount of solitary noise points substantiates the importance of the pixel-attention discriminator in de-snowing.

More noise points are observed in the scenario without SSIM loss. Apart from the slightly reduced cluster number, other metrics, especially the elevated reachability distance, indicate a breakdown in structural integrity during the translation process. A primary objective of our model is to maintain the crucial objects and environmental elements as effectively as possible, thus affirming the critical role of SSIM loss in our model.

The scenario without depth loss indicates a complete failure in de-snowing, as evidenced by the significant plummeting in all metrics toward noisy and poor clustering. The cause of this failure lies in the unique properties of depth images, which are highly sensitive to non-linear scale changes during the conversion back to point clouds. Consequently, the depth loss forms the cornerstone of our translation model based on depth images.

In the basic CycleGAN model, the mediocre statistics could be interpreted as an utter ineffectiveness in point cloud translation, without managing to preserve the original states either. This result underscores the necessity of all the components in our model for achieving successful translation outcomes.

Finally, when trained on datasets with a substantial domain gap, the model does not yield satisfactory de-snowing performance. This is suggested by the exceedingly high noise number, reachability distances, and low cluster sizes, at least under the same parameter settings as before. The unjustifiably high noise and cluster numbers are the result of poor clustering, which is corroborated by the exceedingly high DBI. This result, derived under extreme conditions, serves to confirm our judicious decision to generate unpaired clear data with filters. However, it does not necessarily suggest that our model lacks generality. Despite this, the model’s robustness against domain gaps does stand as a major limitation of our current translation model.

## 5. Conclusions

In this research, we introduced a GAN-driven model capable of translating LiDAR point cloud data, encompassing both the removal of snow and artificial snow production. Utilizing depth image priors of point clouds, our model was trained on unpaired datasets and supplemented with depth loss and SSIM loss functions to ensure scale and structure consistencies. Furthermore, we crafted a novel discriminator structure with an emphasis on pixels. This feature was integrated alongside the original convolution layers, thereby enhancing the snow removal capability in the vicinity of the ego vehicle. Experiments carried out using authentic snow conditions from the CADC dataset revealed a profound comprehension of snow characteristics and driving scenes as well as exceptional performance in snow removal and snow reproduction. The 3D clustering results from the OPTICS algorithm and their corresponding violin plots evidently prove the successful translation between snow and clear conditions.

LiDAR point cloud processing under snowy conditions has consistently faced the challenge of lacking reliable snow-affected data. Given the difficulty in acquiring paired or quasi-paired data under both snowy and clear conditions, our current model must strike a balance between the strength of translation and model stability, which subsequently leads to domain sensitivity. Moreover, the limited resources of the CADC dataset intensify the adversity for training and testing. To address these limitations, our future goal is to develop the capability to generate high-quality paired data under snowy conditions. This aim is to augment the LiDAR point cloud with snow based on a deep understanding of the driving scene, with the ultimate intention of preserving the original state of the scene to the greatest extent possible.

## Figures and Tables

**Table 1:** Annotation of symbols in the model architecture.

Symbols	Annotations	Symbols	Annotations
A	data flow in the forward cycle	B	data flow in the backward cycle
S	snow conditions	C	clear conditions
Snow A	input dataset with snow conditions	Clear B	input dataset under clear conditions
Snow B	model generated snow results	Clear A	model generated clear results
$G_{SC}$	generator for snow to clear	$G_{CS}$	generator for clear to snow
$D_{A}$	discriminator for judging generated clear results	$D_{B}$	discriminator for judging generated snow results
$D_{nA}$	N-layer Discriminator module in $D_{A}$	$D_{nB}$	N-layer Discriminator module in $D_{B}$
$D_{pA}$	pixel-attention discriminator module in $D_{A}$	$D_{pB}$	pixel-attention discriminator module in $D_{B}$
$Rec_{A}$	reconstructed snow results	$Rec_{B}$	reconstructed clear results

**Table 2:** The 3D clustering metrics from the OPTICS algorithm for ascending snow levels.

	Noise Number	Cluster Number	Reachability Distances Avg.	Size of Clusters Avg.	Inter-Cluster Distances Avg.	Davies-Bouldin Index	Silhouette Score
Slight snowfall	1861	761	0.3336	54.0279	17.6373	4.0110	−0.1653
Normal snowfall	2319	982	0.3737	52.8820	14.2597	5.8451	−0.2246
Fierce snow swirl	2942	1165	0.4166	46.1090	12.3494	8.4671	−0.2772

**Table 3:** The 3D clustering metrics (avg.) from the OPTICS algorithm under scattered snow conditions.

	Noise Number	Cluster Number	Reachability Distances	Inter-Cluster Distances	Size of Clusters	Davies-Bouldin Index	Silhouette Score
CADC	2865.07	964.23	0.4076	51.4262	14.1603	4.0279	−0.3011
De-snow	2689.46	792.23	0.3766	53.9163	15.8366	3.7427	−0.2111
Fake snow	3025.06	1011.04	0.4339	51.1447	12.9028	4.7175	−0.2730

**Table 4:** The 3D clustering metrics (avg.) from the OPTICS algorithm under snow swirls conditions.

	Noise Number	Cluster Number	Reachability Distances	Inter-Cluster Distances	Size of Clusters	Davies-Bouldin Index	Silhouette Score
CADC	2598.17	954.24	0.3805	45.9245	14.9750	5.4138	−0.3688
De-snow	1606.23	639.99	0.3293	47.4392	24.2110	3.9073	−0.3109
Fake snow	3124	998.77	0.4358	44.3044	13.7164	6.0746	−0.3661

**Table 5:** Ablation study based on the de-snow model under mild snow conditions.

	Noise Number	Cluster Number	Reachability Distances	Inter-Cluster Distances	Size of Clusters	Davies-Bouldin Index	Silhouette Score
Proposed model	2689.46	792.23	0.3766	53.9163	15.8366	3.7427	−0.2111
w/o $pixel_{D}$	2882.94	761.25	0.4373	52.4850	16.3089	4.0880	−0.3532
w/o ssim loss	2935.19	772.25	0.4511	53.1700	15.7794	4.1745	−0.3394
w/o depth loss	3879.13	1097.3	0.5597	46.1819	9.9985	5.4330	−0.2990
Basic CycleGAN	2891.98	833.47	0.4863	49.9774	14.0490	5.1194	−0.3638
Domain gap	5031.85	1132.72	0.7663	47.2124	10.1896	7.1288	−0.2825

## Data Availability

Not applicable.

## Abbreviations

The following abbreviations are used in this manuscript:

LiDAR	Light Detection And Ranging
GAN	Generative Adversarial Networks
L-DIG	LiDAR Depth Images GAN
ADS	Autonomous Driving Systems
CADC	Canadian Adverse Driving Conditions
OPTICS	Ordering Points To Identify the Clustering Structure
DBSCAN	Density-Based Spatial Clustering of Applications with Noise
SSIM	Structural Similarity Index
DROR	Dynamic Radius Outlier Removal
DSOR	Dynamic Statistical Outlier Removal
PGM	Polar Grid Map
FOV	Field of View
DBI	Davies-Bouldin Index

## References

[1] Carballo A., Lambert J., Monrroy A., Wong D., Narksri P., Kitsukawa Y., Takeuchi E., Kato S., Takeda K. LIBRE: The multiple 3D LiDAR dataset. Proceedings of the Intelligent Vehicles Symposium (IV).

[2] Pitropov M., Garcia D.E., Rebello J., Smart M., Wang C., Czarnecki K., Waslander S. (2021). Canadian adverse driving conditions dataset. Int. J. Robot. Res..

[3] Diaz-Ruiz C.A., Xia Y., You Y., Nino J., Chen J., Monica J., Chen X., Luo K., Wang Y., Emond M. Ithaca365: Dataset and Driving Perception Under Repeated and Challenging Weather Conditions. Proceedings of the IEEE/CVF Conference on Computer Vision and Pattern Recognition.

[4] Zhang Y., Carballo A., Yang H., Takeda K. (2023). Perception and sensing for autonomous vehicles under adverse weather conditions: A survey. ISPRS J. Photogramm. Remote Sens..

[5] Guo A., Feng Y., Chen Z. LiRTest: Augmenting LiDAR point clouds for automated testing of autonomous driving systems. Proceedings of the 31st ACM SIGSOFT International Symposium on Software Testing and Analysis.

[6] Goodin C., Carruth D., Doude M., Hudson C. (2019). Predicting the Influence of Rain on LIDAR in ADAS. Electronics.

[7] Jokela M., Kutila M., Pyykönen P. (2019). Testing and validation of automotive point-cloud sensors in adverse weather conditions. Appl. Sci..

[8] Charron N., Phillips S., Waslander S.L. De-noising of lidar point clouds corrupted by snowfall. Proceedings of the 2018 15th Conference on Computer and Robot Vision (CRV).

[9] Le M.H., Cheng C.H., Liu D.G. (2023). An Efficient Adaptive Noise Removal Filter on Range Images for LiDAR Point Clouds. Electronics.

[10] Sakaridis C., Dai D., Van Gool L. (2018). Semantic foggy scene understanding with synthetic data. Int. J. Comput. Vis..

[11] Zhang H., Patel V.M. Densely connected pyramid dehazing network. Proceedings of the IEEE Conference on Computer Vision and Pattern Recognition.

[12] Uřičář M., Křížek P., Sistu G., Yogamani S. Soilingnet: Soiling detection on automotive surround-view cameras. Proceedings of the Intelligent Transportation Systems Conference (ITSC).

[13] Yue Z., Xie J., Zhao Q., Meng D. Semi-Supervised Video Deraining With Dynamical Rain Generator. Proceedings of the IEEE/CVF Conference on Computer Vision and Pattern Recognition (CVPR).

[14] Ye Y., Chang Y., Zhou H., Yan L. Closing the Loop: Joint Rain Generation and Removal via Disentangled Image Translation. Proceedings of the IEEE/CVF Conference on Computer Vision and Pattern Recognition (CVPR).

[15] Ancuti C., Ancuti C.O., Timofte R. Ntire 2018 challenge on image dehazing: Methods and results. Proceedings of the IEEE/CVF Conference on Computer Vision and Pattern Recognition (CVPR) Workshops.

[16] Engin D., Genç A., Kemal Ekenel H. Cycle-dehaze: Enhanced cyclegan for single image dehazing. Proceedings of the IEEE Conference on Computer Vision and Pattern Recognition Workshops.

[17] Yang H., Carballo A., Zhang Y., Takeda K. (2023). Framework for generation and removal of multiple types of adverse weather from driving scene images. Sensors.

[18] Uřičář M., Sistu G., Rashed H., Vobecky A., Kumar V.R., Krizek P., Burger F., Yogamani S. Let’s Get Dirty: GAN Based Data Augmentation for Camera Lens Soiling Detection in Autonomous Driving. Proceedings of the IEEE/CVF Winter Conference on Applications of Computer Vision (WACV).

[19] Japan Automotive Research Institute (JARI) Special Environment Proving Ground.

[20] Laboratoire régional des ponts et chaussées Site de Clermont-Ferrand.

[21] Heinzler R., Piewak F., Schindler P., Stork W. (2020). Cnn-based lidar point cloud de-noising in adverse weather. IEEE Robot. Autom. Lett..

[22] Zhu J.Y., Park T., Isola P., Efros A.A. Unpaired image-to-image translation using cycle-consistent adversarial networks. Proceedings of the IEEE International Conference on Computer Vision.

[23] Goodfellow I., Pouget-Abadie J., Mirza M., Xu B., Warde-Farley D., Ozair S., Courville A., Bengio Y. (2020). Generative adversarial networks. Commun. ACM.

[24] Yang H., Carballo A., Takeda K. Disentangled Bad Weather Removal GAN for Pedestrian Detection. Proceedings of the 2022 IEEE 95th Vehicular Technology Conference: (VTC2022-Spring).

[25] Caccia L., Van Hoof H., Courville A., Pineau J. Deep generative modeling of lidar data. Proceedings of the 2019 IEEE/RSJ International Conference on Intelligent Robots and Systems (IROS).

[26] Kurup A., Bos J. (2021). Dsor: A scalable statistical filter for removing falling snow from lidar point clouds in severe winter weather. arXiv.

[27] Bergius J. (2022). LiDAR Point Cloud De-Noising for Adverse Weather. Ph.D. Thesis.

[28] Wang C., Ji M., Wang J., Wen W., Li T., Sun Y. (2019). An improved DBSCAN method for LiDAR data segmentation with automatic Eps estimation. Sensors.

[29] Ankerst M., Breunig M.M., Kriegel H.P., Sander J. (1999). OPTICS: Ordering points to identify the clustering structure. ACM Sigmod Rec..

[30] Schubert E., Gertz M. Improving the Cluster Structure Extracted from OPTICS Plots. Proceedings of the LWDA.

[31] El Yabroudi M., Awedat K., Chabaan R.C., Abudayyeh O., Abdel-Qader I. Adaptive DBSCAN LiDAR Point Cloud Clustering For Autonomous Driving Applications. Proceedings of the 2022 IEEE International Conference on Electro Information Technology (eIT).

[32] Park J.I., Park J., Kim K.S. (2020). Fast and accurate desnowing algorithm for LiDAR point clouds. IEEE Access.

[33] Wang W., You X., Chen L., Tian J., Tang F., Zhang L. (2022). A scalable and accurate de-snowing algorithm for LiDAR point clouds in winter. Remote Sens..

[34] Bijelic M., Gruber T., Ritter W. A benchmark for lidar sensors in fog: Is detection breaking down?. Proceedings of the Intelligent Vehicles Symposium (IV).

[35] Shamsudin A.U., Ohno K., Westfechtel T., Takahiro S., Okada Y., Tadokoro S. (2016). Fog removal using laser beam penetration, laser intensity, and geometrical features for 3D measurements in fog-filled room. Adv. Robot..

[36] Hahner M., Sakaridis C., Bijelic M., Heide F., Yu F., Dai D., Van Gool L. Lidar snowfall simulation for robust 3d object detection. Proceedings of the IEEE/CVF Conference on Computer Vision and Pattern Recognition.

[37] Kim T., Cha M., Kim H., Lee J.K., Kim J. Learning to discover cross-domain relations with generative adversarial networks. Proceedings of the International Conference on Machine Learning, PMLR.

[38] Yi Z., Zhang H., Tan P., Gong M. Dualgan: Unsupervised dual learning for image-to-image translation. Proceedings of the IEEE International Conference on Computer Vision.

[39] Jaw D.W., Huang S.C., Kuo S.Y. (2020). DesnowGAN: An efficient single image snow removal framework using cross-resolution lateral connection and GANs. IEEE Trans. Circuits Syst. Video Technol..

[40] Sallab A.E., Sobh I., Zahran M., Essam N. (2019). LiDAR Sensor modeling and Data augmentation with GANs for Autonomous driving. arXiv.

[41] Sobh I., Amin L., Abdelkarim S., Elmadawy K., Saeed M., Abdeltawab O., Gamal M., El Sallab A. (2018). End-to-End Multi-Modal Sensors Fusion System for Urban Automated Driving. https://openreview.net/forum?id=Byx4Xkqjcm.

[42] Geiger A., Lenz P., Urtasun R. Are we ready for autonomous driving? the kitti vision benchmark suite. Proceedings of the 2012 IEEE Conference on Computer Vision and Pattern Recognition.

[43] Lee J., Shiotsuka D., Nishimori T., Nakao K., Kamijo S. (2022). GAN-Based LiDAR Translation between Sunny and Adverse Weather for Autonomous Driving and Driving Simulation. Sensors.

[44] Zyrianov V., Zhu X., Wang S. (2022). Learning to generate realistic lidar point clouds. Proceedings of the European Conference on Computer Vision.

[45] Mertan A., Duff D.J., Unal G. (2022). Single image depth estimation: An overview. Digit. Signal Process..

[46] Eigen D., Puhrsch C., Fergus R. (2014). Depth map prediction from a single image using a multi-scale deep network. Adv. Neural Inf. Process. Syst..

[47] Kokoska S., Zwillinger D. (2000). CRC Standard Probability and Statistics Tables and Formulae.

